# Morphology-Evolving Colorimetric Thin-Film Sensor for Visual Detection of Hypochlorous Acid

**DOI:** 10.3390/s26072082

**Published:** 2026-03-27

**Authors:** Yasumasa Kanekiyo, Takumi Kato, Emi Sakai

**Affiliations:** School of Regional Innovation and Social Design Engineering, Kitami Institute of Technology, 165 Koen-cho, Kitami 090-8507, Hokkaido, Japan

**Keywords:** hypochlorous acid, hypochlorite, thin film, colorimetric sensor, pattern change, dye

## Abstract

**Highlights:**

**What are the main findings?**

- A morphology-evolving thin-film sensor was developed that converts HClO-induced charge-state changes into distinct color and pattern transitions (blue X → red circle).
- The dual-mode response enables both visual discrimination and semi-quantitative estimation of HClO concentration and successfully tracks disinfectant decomposition under UV irradiation.

**What are the implications of the main findings?**

- Pattern-coupled colorimetric sensing enhances naked-eye readability compared to conventional color-only assays.
- The integration of geometric pattern inversion with chromatic information provides improved robustness for practical, image-based chemical sensing in real-world environments.

**Abstract:**

Hypochlorous acid (HClO) is widely used as a low-cost and effective disinfectant; however, its instability under heat and light necessitates simple and reliable monitoring methods. Herein, we report a morphology-evolving thin-film colorimetric sensor that enables intuitive visual detection of HClO through simultaneous color and pattern transitions. The sensor integrates two polymer films with distinct charge-state response behaviors, patterned in X-shaped and circular geometries on a single substrate. Upon exposure to HClO, chlorine-induced modification of amide and amine groups alters the surface charge states, thereby switching the adsorption preference for anionic and cationic dyes. This mechanism results in a pronounced transformation from a blue X-shaped motif to a red circular pattern, enabling direct visual discrimination between different HClO concentrations. Quantitative analysis of RGB values confirmed semi-quantitative detection in the sub-millimolar to millimolar range. The sensor exhibited a linear response in the range of 0–3 mM (R^2^ > 0.979) with a limit of detection of 0.103 mM. The sensor further demonstrated practical applicability by tracking photodecomposition of a commercial disinfectant. This work demonstrates pattern-coupled colorimetric sensing as a straightforward, user-friendly approach for HClO monitoring.

## 1. Introduction

The COVID-19 pandemic has heightened global awareness of the risks posed by infectious diseases. Even in the post-pandemic era, the importance of preparedness against a broad range of viral and bacterial infections remains widely recognized. Disinfection of potentially contaminated surfaces—such as hands, doorknobs, and tabletops—is among the most effective measures for interrupting transmission pathways [1]. Hypochlorous acid and hypochlorite (HClO) have been reported to exhibit potent antiviral and antimicrobial activity against diverse viruses and bacteria [2,3,4,5]. Because HClO can be generated simply through the electrolysis of sodium chloride, it represents a low-cost and scalable alternative to conventional alcohol-based disinfectants [6]. Additional advantages include its low allergenicity and its efficacy against alcohol-resistant pathogens such as norovirus [7,8]. However, HClO readily decomposes, particularly at elevated temperatures and upon light exposure, necessitating regular monitoring of its concentration to ensure disinfection efficacy [9,10].

HClO is widely used as a disinfectant due to its strong antimicrobial activity and low toxicity. In practical applications, HClO-based disinfectants are typically used at concentrations ranging from several tens to hundreds of ppm (sub-millimolar to millimolar levels). The speciation of chlorine is pH-dependent, and HClO is the dominant and more reactive species under weakly acidic conditions (pH 5–6.5), which are commonly used in disinfectant formulations.

In household settings, HClO concentration is commonly measured using test strips based on the DPD method, in which *N*,*N*-diethyl-p-phenylenediamine (DPD) is oxidized to produce a characteristic pink coloration [11]. While widely used, the DPD method has a significant limitation: subtle changes in color intensity are difficult to distinguish visually. The reaction product is typically a pale pink hue, and the incremental differences between concentration levels are often ambiguous, especially in turbid samples or for users with color-vision deficiencies [12]. Moreover, the DPD method is vulnerable to interference from other oxidizing agents, potentially resulting in false-positive readings [13]. The reagent itself also undergoes degradation upon exposure to oxygen, light, or humidity, requiring careful handling and storage to maintain accuracy [14]. Therefore, there is a need for alternative sensing strategies that provide clear, unambiguous, and visually intuitive readouts, enabling reliable on-site monitoring of HClO concentration without interference from other oxidizing agents. Recently, various sensing strategies for HClO detection, including fluorescent, colorimetric, and electrochemical approaches, have been developed [15,16].

Our research group previously developed thin-film-based colorimetric sensing platforms that exhibit exceptionally clear color transitions [17,18,19,20]. More recently, we have developed a method for conjugating lactic acid-responsive polymers to cotton fabric using photomasks and stencils of various shapes and have succeeded in creating a fabric sensor that allows changes in the pattern to be visually and intuitively observed [21]. These systems operate through a straightforward mechanism: reaction with a target analyte alters the charge state of the polymer, which in turn modulates the adsorption of ionic dyes on the film surface. The fabrication process is simple, does not require complex organic syntheses, and offers broad freedom in the selection of dyes. We hypothesized that this sensing principle could be adapted for HClO detection. Furthermore, we anticipated that patterning two thin-film chemistries with different charge-state response behaviors—each in a distinct geometric motif on the same substrate and each selectively stained with a different dye—would enable simultaneous color and pattern changes upon exposure to HClO.

Colorimetric sensors based solely on color intensity changes are generally suitable for quantitative measurements, as their responses can be readily digitized through spectroscopic or image-based analysis. However, such systems typically require strictly controlled illumination conditions to maintain accuracy, since variability in lighting represents a major source of measurement error in AI-based visual sensing [22]. Under fluctuating illumination, quantitative reliability can deteriorate significantly. In contrast, sensors that exhibit both color and pattern changes provide enhanced visual detectability and user accessibility, as pattern recognition relies primarily on geometric and contrast-based features rather than absolute spectral information [23]. Consequently, color–pattern dual-mode sensors are inherently more robust against illumination variations and may offer greater reliability in practical, real-world environments, whereas color-only sensors remain advantageous for precise quantification under well-controlled conditions.

In this work, we present a dual-mode colorimetric sensor for HClO that achieves simultaneous color and pattern transitions by integrating two differently responsive polymer films patterned on a single substrate. This design provides naked-eye readout with improved robustness against illumination variations while still allowing semi-quantitative analysis via RGB (Red-Green-Blue) or spectroscopic methods.

## 2. Materials and Methods

### 2.1. Reagents and Materials

**Scheme 1:**
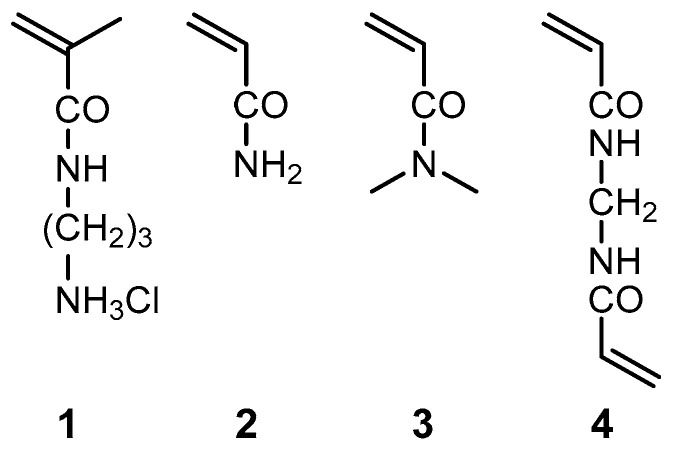
Chemical structures of the monomers used for fabrication of the thin-film sensor.

**Scheme 2:**
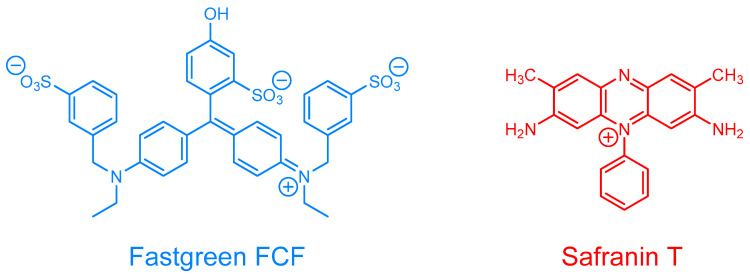
Chemical structures of the anionic and cationic dyes used for sensor coloration.

The chemical structures of the monomers and anionic dyes are presented in Scheme 1 and Scheme 2, respectively. The primary amine monomer (*N*-(3-aminopropyl)methacrylamide hydrochloride, **1**), acrylamide (**2**), crosslinker (*N*,*N*′-methylenebisacrylamide, **4**), initiator (2,2′-Azobis(2-methylpropionamidine) dihydrochloride, AAPH), Dimethyl sulfoxide (DMSO), and sodium hypochlorite pentahydrate (NaClO·5H_2_O) were purchased from FUJIFILM Wako Pure Chemicals (Osaka, Japan). *N*,*N*-dimethylacrylamide (**3**), Fastgreen FCF (FG), and Safranin T (SF) were purchased from Tokyo Chemical Industry (Tokyo, Japan). The reagents for preparing the buffer solutions [2-{4-(2-hydroxyethyl)-1-piperazinyl}ethanesulfonic acid (HEPES)] were purchased from Dojindo Laboratories (Kumamoto, Japan). All chemicals were purchased as reagent grade and used as received, without further purification. A slide glass (MAS coat, SMAS-01) was obtained from Matsunami Glass Industry (Osaka, Japan). The slide surface is modified with hydrophilic amino groups.

### 2.2. Fabrication of Hypochlorous Acid-Responsive Thin Films

**Scheme 3:**
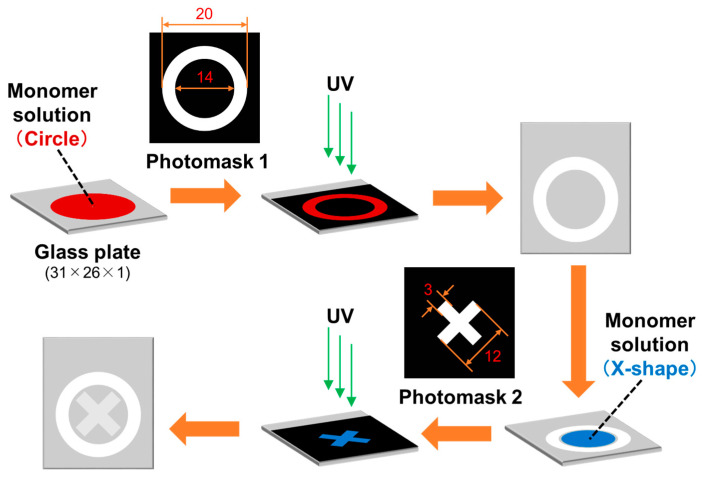
Schematic illustration of the fabrication process of the dual-pattern thin-film sensor (dimensions in mm).

An outline of the sample preparation process is illustrated in Scheme 3. Two monomer solutions were prepared with compositions listed in Table 1, which summarizes the monomer formulations used for the circular and X-shaped patterned regions, respectively. As the first step, the monomer solution for circular pattern was poured onto the slide glass using a micropipette (80 μL) and covered with an acrylic plate. Polymerization was conducted by irradiating the sandwiched monomer solution with UV light (365 nm) using a handy UV lamp LUV-6 (AS ONE, Osaka, Japan) equipped with a 6 W discharge tube at 20–25 °C for 1 h. To avoid oxygen interference, the procedure was conducted in a glove box under a nitrogen atmosphere. After removing the acrylic plate from the slide glass, the resulting thin films were washed with water for 10 min and air-dried. As the second step, the monomer solution for X-shaped pattern was poured onto the surface of the slide glass using a micropipette (65 μL) and covered with an acrylic plate. Polymerization was carried out in the same manner as in the first step. The difference in the volume of the monomer solutions (80 μL for the circular pattern and 65 μL for the X-shaped pattern) was introduced to ensure complete and uniform coverage of each patterned area during fabrication. The resulting thin films were visually confirmed to form uniform and well-defined patterns with good reproducibility. Therefore, the difference in volume did not significantly affect the sensing behavior, which is primarily governed by the chemical composition of each polymer film. After removing the acrylic plate from the slide glass, the resulting thin films were washed with 10 mM sodium carbonate solution and water for 10 min each, and air-dried.

### 2.3. Preparation of Hypochlorous Acid Solution

First, a sodium hypochlorite stock solution was prepared at an initial concentration of approximately 100 mM. The exact concentration of hypochlorite in the stock solution was determined by measuring the absorbance at 292 nm (A292) of a solution diluted 100-fold with water using the following Equation (1):

[NaClO] = (A292/ε) × 10^5^ (1)

where ε represents the molar absorptivity of hypochlorite, and a value of 357 M^−1^ cm^−1^ was used in this study, as reported in the literature [24]. According to the Beer–Lambert law, the absorbance is proportional to the concentration of hypochlorite. The concentration was therefore calculated using the molar absorptivity without the need for an external calibration curve.

Subsequently, a slightly acidic HClO solution was prepared by neutralizing hypochlorite with 0.9 equivalent of HCl as follows. The required amount of sodium hypochlorite stock solution and 90 μL of 1 M hydrochloric acid were taken into a 10 mL volumetric flask and diluted with water. The required amount of the stock solution was adjusted so that the total concentration of HClO after dilution was 10 mM. When measuring the sensor response to HClO, this solution was diluted with water appropriately to a predetermined HClO concentration.

### 2.4. Response to Hypochlorous Acid

The sensor was immersed in 30 mL of aqueous solutions containing HClO (0–3 mM) at 25 °C for 10 min. Subsequently, the sensor was washed with water and air-dried. The dried sensor was then immersed in aqueous dye solutions at 25 °C for 5–60 min. The dye solution contained 20 μM FG, 200 μM SF, and 10 mM HEPES (pH 7.4). After washing with water and drying in air, sample photographs were taken and the ultraviolet-visible (UV-vis) absorption spectra of the thin films were measured by the diffuse-reflection method at normal incidence using an integrated sphere. RGB values were obtained from the sample photographs using the RGB value acquisition software “IRO-DORI” software [25].

### 2.5. Apparatus

The aqueous solutions were prepared with purified distilled water using a WG202 system (Yamato Scientific Co., Ltd., Tokyo, Japan). The pH values were determined using a pH meter (827 pH Lab, Metrohm, Herisau, Switzerland). The photographs of the samples were captured using a digital camera (COOLPIX P310, Nikon, Tokyo, Japan). UV–vis absorption spectra were obtained using a spectrophotometer (V-650, JASCO, Tokyo, Japan) equipped with an integrating sphere (ISV-722, JASCO, Tokyo, Japan). ATR-FTIR spectra were recorded using an FT/IR-4600 spectrometer (JASCO, Tokyo, Japan) equipped with an ATR-PRO ONE X accessory with a ZnSe prism.

## 3. Results

### 3.1. Color and Pattern Changes Induced by HClO

**Figure 1:**
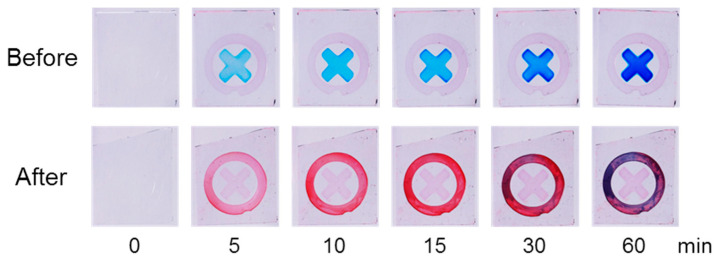
Visual color changes in the sensor before (**top**) and after (**bottom**) immersion in 2 mM HClO solution for 10 min, followed by dye staining.

The sensor was stained with a dye solution containing Fast Green (FG, blue) and Safranin T (SF, red) (Figure 1). Before exposure to HClO, a blue X-shaped pattern gradually emerged on the sensor surface with increasing staining time, while the circular region remained nearly colorless. After immersion in HClO solution followed by dye staining, the coloration pattern changed markedly: the blue X-shaped pattern weakened or disappeared, and a red circular pattern became visible.

**Figure 2:**
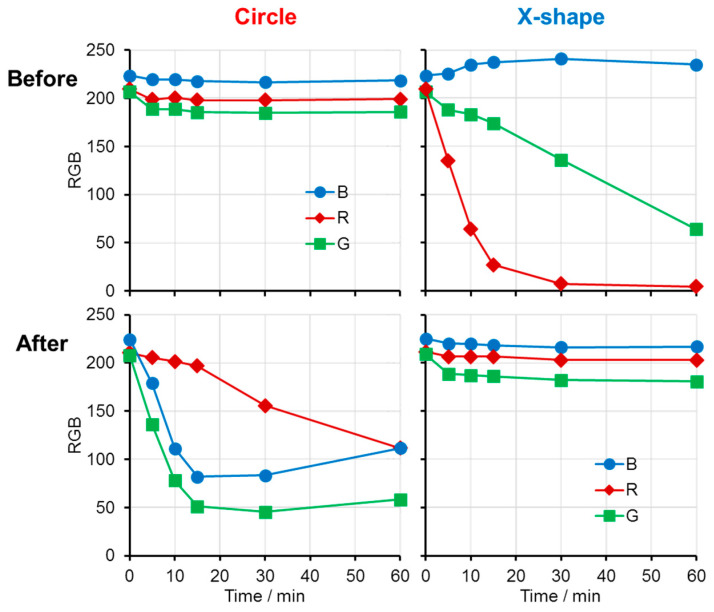
Changes in RGB values of the sensor before (**top**) and after (**bottom**) immersion in 2 mM HClO solution for 10 min, followed by dye staining. Left: circular area; right: X-shaped area. RGB values are expressed as unitless parameters (0–255 scale).

To quantitatively evaluate the response, RGB values were extracted from sample photographs and plotted as a function of staining time (Figure 2). In the circular area, only minor changes in RGB values were observed prior to HClO exposure, whereas substantial decreases in the G and B values were observed after HClO treatment, corresponding to red coloration. In contrast, the X-shaped region exhibited a pronounced change in the R value before HClO exposure but showed little variation after HClO treatment. These results indicate that HClO exposure induces distinct and measurable changes in the optical properties of each patterned region.

To assess the chemical reactions induced by HClO on the polymer, ATR-FTIR spectra were measured in the dry state. The FTIR spectra (Figure S4) revealed distinct differences between the two regions. In the X region, the N–H stretching (~3300 cm^−1^) and bending (~1600 cm^−1^) bands decreased after HClO treatment, while the C–H (~2900 cm^−1^) and amide I (~1650 cm^−1^) bands remained largely unchanged. In the circular region, only minor spectral changes were observed; the amide I and C–H bands remained essentially unchanged, while a slight increase around ~1600 cm^−1^ was detected.

### 3.2. Dependence of Sensor Response on HClO Concentration

**Figure 3:**
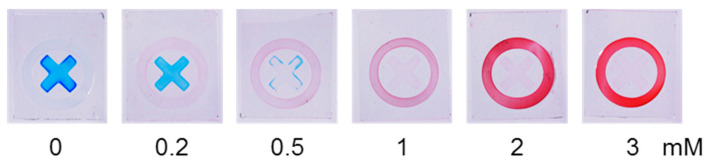
Concentration-dependent pattern and color changes in the sensor after immersion in HClO solutions (0–3 mM) for 10 min, followed by dye staining for 15 min.

The concentration-dependent response of the sensor to HClO was investigated (Figure 3). In the absence of HClO, only the blue X-shaped pattern was observed. As the HClO concentration increased, the X-shaped pattern gradually faded and nearly disappeared at 1 mM. Simultaneously, a red circular pattern emerged and intensified with increasing HClO concentration, becoming faintly visible even at 0.2 mM. At higher concentrations, only a dark red circular pattern remained. These clearly observable transitions enabled visual discrimination of HClO concentration.

**Figure 4:**
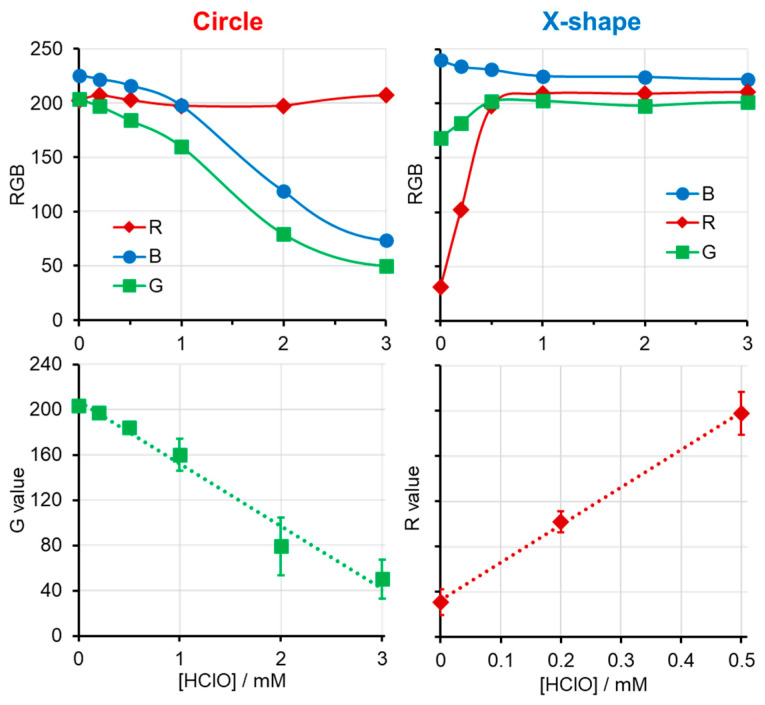
Relationship between RGB values and HClO concentration. Top: changes in RGB values in the circular (**left**) and X-shaped (**right**) regions. Bottom left: relationship between the G value and HClO concentration in the range of 0–3 mM (circular region). Bottom right: calibration plot based on the R value in the range of 0–0.5 mM (X-shaped region). Linear fitting was performed in these ranges (dotted lines), with correlation coefficients (R^2^) of 0.979 and 0.999, respectively. The limits of detection (LOD) were estimated to be 0.136 mM and 0.103 mM, respectively, based on 3σ/slope. Error bars represent standard deviations obtained from repeated measurements (n = 10). RGB values are expressed as unitless parameters (0–255 scale).

RGB values for both patterned regions were plotted against HClO concentration (Figure 4). In the circular area, all RGB values were approximately 200 at zero HClO concentration, consistent with a nearly colorless appearance. With increasing HClO concentration, the red coloration intensified, leading to significant decreases in the G and B values. In the X-shaped region, the R value was initially low due to blue coloration, but increased progressively as the blue color faded. At sufficiently high HClO concentrations, both regions became nearly colorless or uniformly red, and RGB values approached similar magnitudes. To evaluate the analytical performance of the sensor, calibration plots were constructed based on RGB values. As shown in Figure 4, the G value in the circular region exhibited a concentration-dependent decrease over a wide range (0–3 mM), with a correlation coefficient (R^2^) of 0.979. In addition, the R value in the X-shaped region showed a linear increase in the low concentration range (0–0.5 mM), with a correlation coefficient (R^2^) of 0.999. The limit of detection (LOD) was estimated based on 3σ/slope, where σ was determined from repeated measurements of the blank signal (n = 10). The LOD values were calculated to be 0.136 mM and 0.103 mM for the G and R values, respectively. These results indicate that the sensor enables both wide-range detection and sensitive quantification in the low concentration region.

The effects of reaction time and pH on the sensor response were investigated under controlled conditions. Upon exposure to 2 mM HClO, the sensor exhibited a clear time-dependent response. The initial blue X-shaped pattern gradually changed through intermediate states and eventually transformed into a red circular pattern with increasing reaction time (Figure S1). In addition, the sensor response was found to depend on pH. Under otherwise identical conditions, a more pronounced color and pattern change was observed at pH 6.5 than at pH 7.5 (Figure S2). At pH 7.5, the response was weaker and proceeded more slowly compared to that at pH 6.5.

The selectivity of the sensor was further evaluated using representative oxidizing agents, including H_2_O_2_ and tert-butyl hydroperoxide, at 2 mM. As shown in Figure S3, no significant color or pattern change was observed for these oxidants, and the original blue X-shaped pattern was retained. In contrast, HClO induced a distinct red circular pattern. These results indicate that the sensor response is not triggered by general oxidation, but is more specific to HClO.

**Figure 5:**
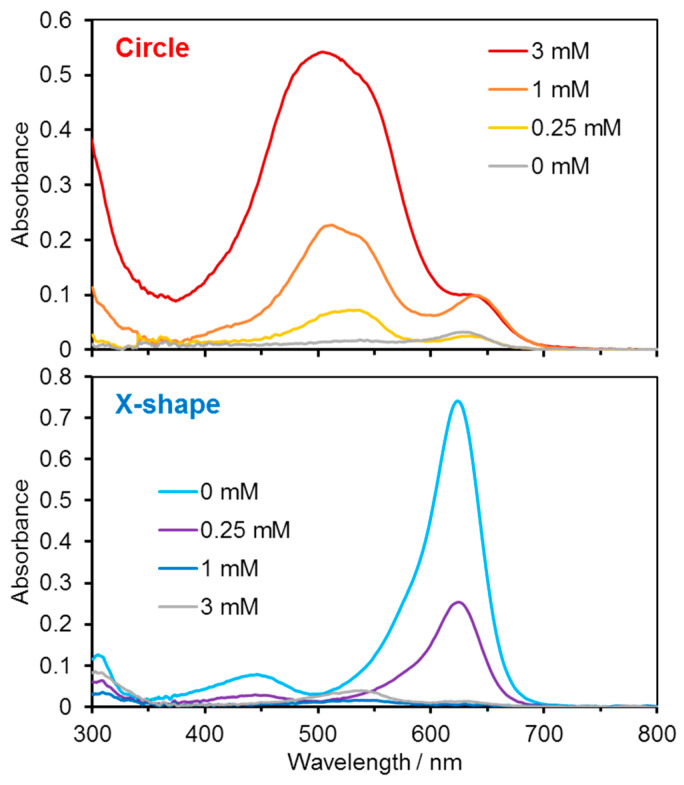
Diffuse-reflectance UV–vis spectral changes of (**top**) the circular area and (**bottom**) the X-shaped area as a function of HClO concentration. Absorbance is given in arbitrary units.

Diffuse-reflectance UV–vis spectra were further measured to obtain more quantitative information (Figure 5). The absorption peak around 500 nm in the circular region increased with increasing HClO concentration, whereas the peak around 630 nm in the X-shaped region decreased correspondingly. Calibration plots constructed from these absorbance values demonstrated a concentration-dependent response, indicating that HClO levels can be estimated based on optical measurements.

### 3.3. Monitoring Decomposition of a Commercial Disinfectant

**Figure 6:**
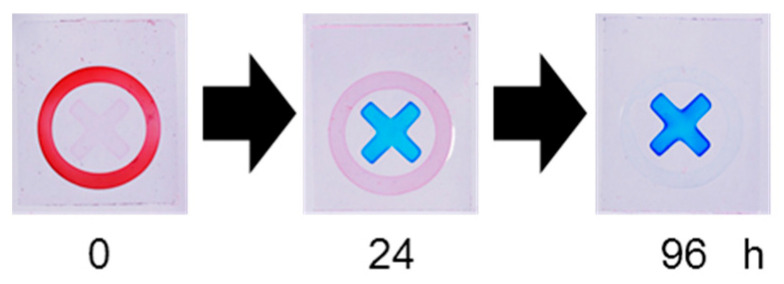
Monitoring of HClO decomposition in a commercially available disinfectant under UV irradiation (365 nm). The sensor response was recorded after 0, 24, and 96 h of irradiation.

To evaluate practical applicability, the sensor was applied to a commercially available disinfectant (Figure 6). Upon exposure to the fresh disinfectant solution, a red circular pattern appeared, indicating a high HClO concentration. The disinfectant was then transferred to a transparent glass bottle and irradiated with 365 nm UV light at 25 °C under stirring to accelerate HClO decomposition.

After 24 h of UV irradiation, the red circular pattern weakened, and a pale blue X-shaped pattern became visible. After 96 h, a marked blue X-shaped pattern was observed, indicating near-complete loss of HClO. According to the product label, the initial available chlorine concentration was 200 ppm (approximately 3 mM HClO), consistent with the sensor response observed in Figure 3. After 24 h, the response corresponded approximately to 0.2–0.5 mM HClO, and after 96 h, the signal indicated a near-zero concentration. To further support the applicability of the sensor for real samples, the response of the sensor to disinfectant samples was compared with a commercially available colorimetric method (orthotolidine method). As shown in Figure S5, the sensor response exhibited consistent changes corresponding to the decrease in HClO concentration over time. After 24 h of irradiation, the HClO concentration was estimated to be 0.2–0.5 mM, which is consistent with the results described above. These results demonstrate that our sensor can visually track HClO degradation in practical disinfectant samples.

## 4. Discussion

**Scheme 4:**
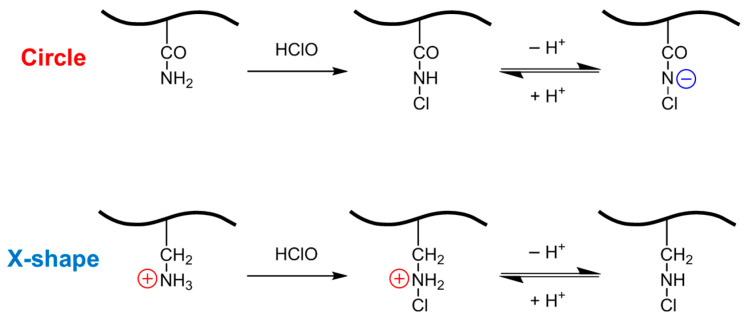
Proposed reaction mechanisms of the polymer films with HClO in each patterned area, showing chloramide formation in the circular region and chloramine formation in the X-shaped region.

The observed color and pattern transitions originate from HClO-induced modulation of the surface charge states of the two polymer films (Scheme 4). The X-shaped region was fabricated using cationic monomers and is initially positively charged, enabling electrostatic adsorption of the anionic dye (FG). In contrast, the circular region was composed of neutral monomers and exhibited minimal interaction with either dye prior to HClO exposure.

Upon treatment with HClO, chemical modification of functional groups alters the charge states in each region. In the circular region (amide-rich), HClO reacts with amide groups to form N-chloramides [26,27], which exhibit markedly increased acidity [28], thereby facilitating deprotonation at neutral pH. This generates negative charges that preferentially adsorb the cationic Safranin T, resulting in red coloration. In the X-shaped region (amine/ammonium-rich), formation of chloramines reduces the basicity of the nitrogen sites [29,30,31]. This diminishes positive charge density and thereby suppresses the adsorption of the anionic Fast Green FCF, leading to the loss of blue coloration.

The spectroscopic results of FTIR suggest that the chemical modification induced by HClO differs between the two regions. In the X region, the decrease in N–H bands indicates modification of primary amine groups, while the unchanged backbone signals imply that the reaction is localized at the functional groups. In the circular region, the limited spectral changes are consistent with the relatively low reactivity of amide groups, with only subtle surface modification occurring. The difference in staining behavior is therefore attributed to variations in the availability and characteristics of dye adsorption sites created by these chemical changes. The observations are consistent with the formation of chloramine species in the amine-containing region and N-chloroamide species in the circular region, although this assignment remains indirect. More direct characterization, such as XPS analysis of the N 1s region, would be helpful to further confirm the presence of N–Cl species.

The complementary charge-state switching mechanism enables the dual-mode response in which one pattern fades while the other emerges. Compared with conventional color-only systems, this coupled color–pattern transition enhances visual contrast and improves interpretability. Furthermore, because the sensing response involves geometric pattern inversion rather than solely intensity variation, the platform may provide improved robustness in practical imaging environments where illumination conditions are not strictly controlled.

The sensing process is based on an irreversible chemical modification induced by HClO, indicating that the sensor is intended for single-use applications. Notably, samples stored for up to five years under ambient dark conditions exhibited similar responses to freshly prepared samples, demonstrating excellent long-term storage stability prior to use.

Previously reported colorimetric methods typically exhibit limits of detection in the sub-millimolar to micromolar range [15,16], depending on the sensing mechanism. In comparison, the present sensor provides sufficient sensitivity (LOD = 0.103 mM) while offering a simple, stable, and visually intuitive detection platform. Commercial disinfectants typically contain chlorine at around 200 ppm (approximately 3 mM HClO). This concentration is much higher than the limits of detection obtained in this study, indicating that the sensor is sufficiently sensitive for practical disinfection applications.

The observed time-dependent response indicates that the interaction between HClO and the polymer film proceeds progressively, leading to a gradual change in surface properties and dye adsorption behavior. The pH dependence of the response can be explained by the pH-dependent speciation of chlorine. HClO is the dominant species under weakly acidic conditions, whereas the fraction of hypochlorite ion (ClO^−^) increases at higher pH. Since HClO is more reactive toward nitrogen-containing functional groups in the polymer, the sensor response is more efficient at lower pH. These results suggest that both the rate and extent of the sensor response are governed by chlorine speciation.

Overall, the combination of selective charge modulation, dual-dye staining, and patterned film architecture underlies the dramatic morphology-evolving response observed in this study and supports the utility of this approach for practical HClO monitoring.

## 5. Conclusions

In this study, we developed a thin-film–based colorimetric sensor capable of detecting hypochlorous acid through simultaneous color and pattern transitions. By integrating two polymer films with distinct charge-state response behaviors and staining them with complementary ionic dyes, the sensor exhibited a pronounced transformation from a blue X-shaped motif to a red circular pattern upon exposure to HClO. Quantitative evaluation using RGB analysis confirmed semi-quantitative detection of HClO within a practical concentration range.

The sensor further demonstrated applicability in monitoring the photodecomposition of a commercially available disinfectant, clearly visualizing the decline in available chlorine under UV irradiation. Beyond its naked-eye readability, the color–pattern dual-mode strategy offers intrinsic robustness against illumination variability, a major source of error in AI-based image analysis of conventional colorimetric systems. By combining geometric pattern recognition with chromatic information, this platform balances quantitative capability with practical reliability, highlighting its potential for real-world, on-site chemical sensing applications.

However, further validation in real water samples is required to assess potential matrix effects and improve quantitative accuracy. Future work will focus on expanding applicability and refining sensor performance for practical applications.

## Figures and Tables

**Table 1:** Monomer compositions (unit: mM) ^1^.

Pattern	Amine Monomer (1)	Acrylamide (2)	DMAA (3)	Crosslinker (4)
Circle	0	950	0	75
X-shape	40	0	910	100

^1^ Initiator: AAPH (50 mM), solvent: H_2_O/DMSO = 1:1 (*v*/*v*).

## Data Availability

The data presented in this study are available from the corresponding author upon reasonable request.

## Supplementary Materials

The following supporting information can be downloaded at: https://www.mdpi.com/article/10.3390/s26072082/s1, Figure S1: Time-dependent color and pattern changes of the sensor upon exposure to 2 mM HClO, followed by dye staining under identical conditions. The original blue X-shaped pattern gradually changes through intermediate states and eventually transforms into a red circular pattern with increasing reaction time, indicating progressive reaction with HClO; Figure S2: pH-dependent color and pattern changes of the sensor upon exposure to HClO under identical conditions, followed by dye staining. A more pronounced response was observed at pH 6.5 (left) compared to pH 7.5 (right), indicating that the sensor response is strongly influenced by the pH-dependent speciation of chlorine; Figure S3: Photographs of the sensor after treatment with 2 mM HClO, H_2_O_2_, and tert-butyl hydroperoxide (t-BuOOH) under identical conditions. Only HClO induced a distinct color and pattern transition, whereas the other oxidants retained the original blue X-shaped pattern, indicating negligible response; Figure S4: ATR-FTIR spectra of the polymer films before and after exposure to 2 mM HClO for 10 min in the X-shaped region (top) and the circular region (bottom). Changes in characteristic absorption bands were observed after HClO treatment in both regions, indicating chemical modification of the polymer. The differences in spectral features between the two regions are consistent with their distinct response behaviors toward HClO; Figure S5: Photographs of a commercially available disinfectant solution before (0 h) and after (24 h) UV irradiation (365 nm, 25 °C). The HClO concentration was evaluated after 100-fold dilution using a conventional colorimetric method (orthotolidine method).

## Abbreviations

The following abbreviations are used in this manuscript:

AAPH	2,2′-Azobis(2-methylpropionamidine) dihydrochloride
DMSO	Dimethyl sulfoxide
DPD	N,N-diethyl-p-phenylenediamine
FG	Fastgreen FCF
HClO	Hypochlorous acid
HEPES	2-{4-(2-hydroxyethyl)-1-piperazinyl}ethanesulfonic acid
NaClO	Sodium hypochlorite
RGB	Red–Green–Blue
SF	Safranin T
UV-vis	Ultraviolet-visible
