# Supplementary material for: Morphology-Evolving Colorimetric Thin-Film Sensor for Visual Detection of Hypochlorous Acid
Source: Sensors (Basel). 2026 Mar 27;26(7):2082. doi: 10.3390/s26072082 (PMC13074471; doi:10.3390/s26072082)
Supplement: Supplementary file 1 [file sensors-26-02082-s001.zip › sensors-4213313-supplementary.pdf]

Supplementary Materials

Morphology-Evolving Colorimetric Thin-Film Sensor for Visual Detection of Hypochlorous Acid

Yasumasa Kanekiyo* and Emi Sakai

School of Regional Innovation and Social Design Engineering, Kitami Institute of Technology, 165 Koen-cho, Kitami, Hokkaido 090-8507, Japan

Corresponding author:

Yasumasa Kanekiyo

E-mail: kanekiyo@mail.kitami-it.ac.jp

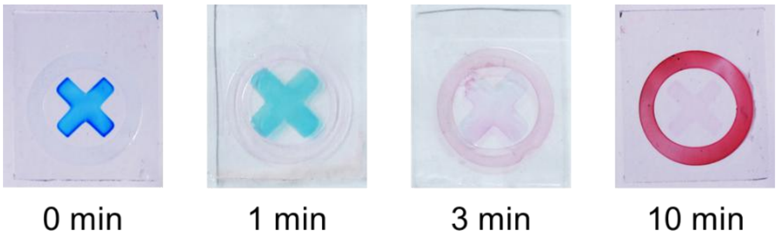

Figure S1. Time-dependent color and pattern changes of the sensor upon exposure to 2 mM HClO, followed by dye staining under identical conditions. The original blue X-shaped pattern gradually changes through intermediate states and eventually transforms into a red circular pattern with increasing reaction time, indicating progressive reaction with HClO.

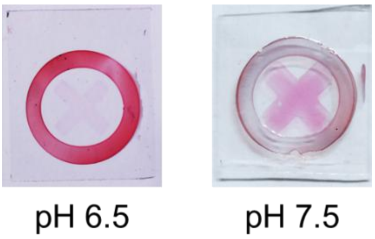

Figure S2. pH-dependent color and pattern changes of the sensor upon exposure to HClO under identical conditions, followed by dye staining. A more pronounced response was observed at pH 6.5 (left) compared to pH 7.5 (right), indicating that the sensor response is strongly influenced by the pH-dependent speciation of chlorine.

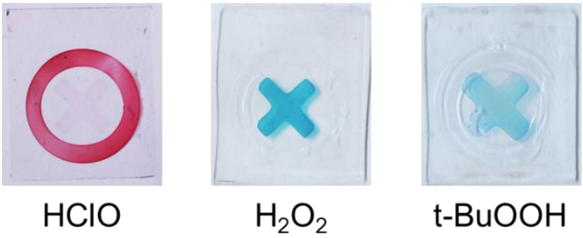

Figure S3. Photographs of the sensor after treatment with 2 mM HClO, H_2O_2, and tert-butyl hydroperoxide (t-BuOOH) under identical conditions. Only HClO induced a distinct color and pattern transition, whereas the other oxidants retained the original blue X-shaped pattern, indicating negligible response.

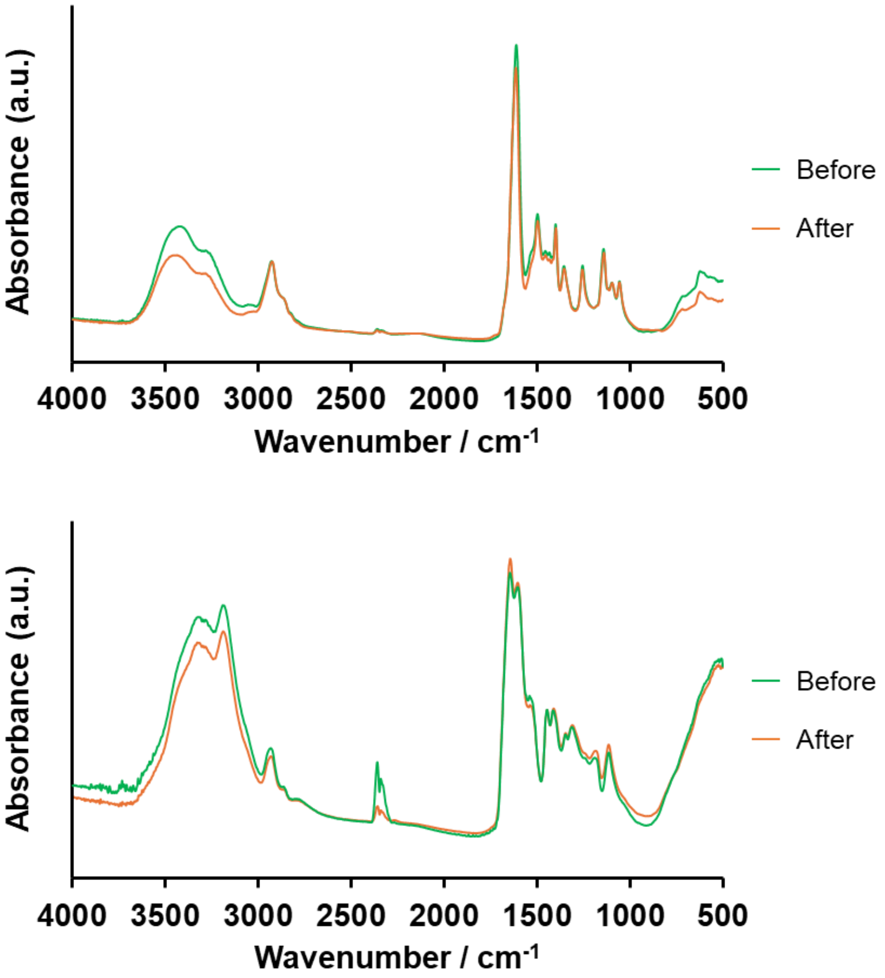

Figure S4. ATR-FTIR spectra of the polymer films before and after exposure to 2 mM HClO for 10 min in the X-shaped region (top) and the circular region (bottom). Changes in characteristic absorption bands were observed after HClO treatment in both regions, indicating chemical modification of the polymer. The differences in spectral features between the two regions are consistent with their distinct response behaviors toward HClO.

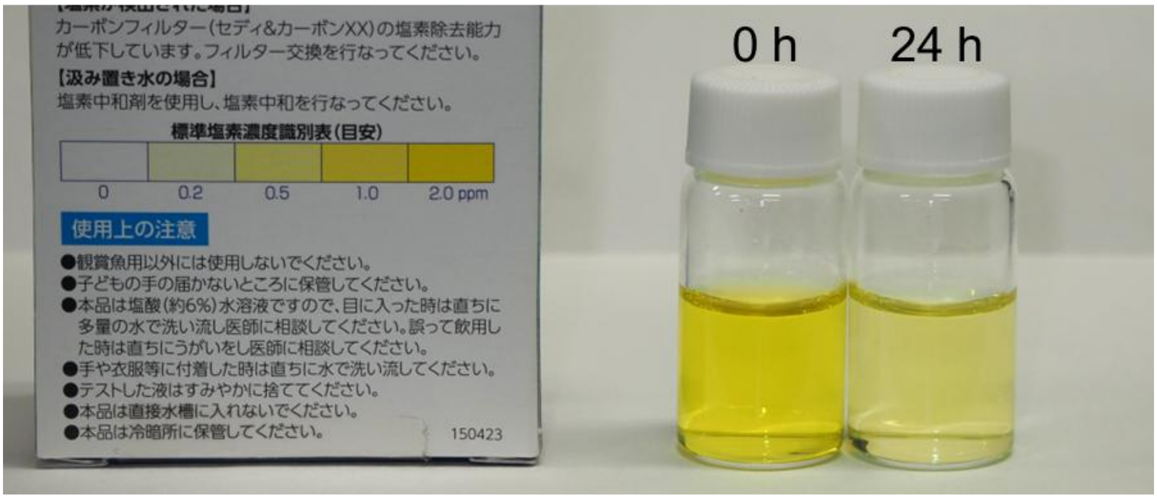

Figure S5. Photographs of a commercially available disinfectant solution before (0 h) and after (24 h) UV irradiation (365 nm, 25 °C). The HClO concentration was evaluated after 100-fold dilution using a conventional colorimetric method (orthotolidine method).
